# The contribution of frequency-specific activity to hierarchical information processing in the human auditory cortex

**DOI:** 10.1038/ncomms5694

**Published:** 2014-09-02

**Authors:** L. Fontolan, B. Morillon, C. Liegeois-Chauvel, Anne-Lise Giraud

**Affiliations:** 1 Department of Neuroscience, University of Geneva, Biotech Campus, 9, Chemin des Mines, Geneva 1211, Switzerland; 2 Department of Psychiatry, Columbia University Medical Center, New York, New York 10032, USA; 3 INSERM U1106—Institut de Neurosciences des Systèmes, Université Aix-Marseille, Marseille 13005, France; 4 These authors contributed equally to this work

## Abstract

The fact that feed-forward and top-down propagation of sensory information use distinct frequency bands is an appealing assumption for which evidence remains scarce. Here we obtain human depth recordings from two auditory cortical regions in both hemispheres, while subjects listen to sentences, and show that information travels in each direction using separate frequency channels. Bottom-up and top-down propagation dominates in *γ*- and *δ*–*β* (<40 Hz) bands, respectively. The predominance of low frequencies for top-down information transfer is confirmed by cross-regional frequency coupling, which indicates that the power of *γ*-activity in A1 is modulated by the phase of *δ*–*β* activity sampled from association auditory cortex (AAC). This cross-regional coupling effect is absent in the opposite direction. Finally, we show that information transfer does not proceed continuously but by time windows where bottom-up or top-down processing alternatively dominates. These findings suggest that the brain uses both frequency- and time-division multiplexing to optimize directional information transfer.

## Abstract (web summary)

Sensory processing relies on information transfer in cortical hierarchies. Using depth recordings of neural activity obtained while individuals with epilepsy listen to spoken sentences, the authors show that ascending and descending information is propagated between cortical regions through distinct neural frequencies.

## Graphical abstract

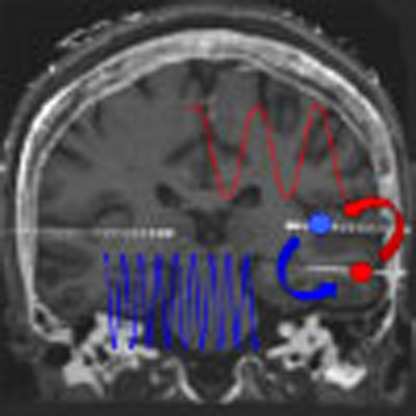

A popular view of brain functioning is that the central neural system minimizes its reaction to environmental stimuli by predicting probable events and inferring their most probable causes^1^,^2^,^3^. This mechanism ensures that reactions are appropriate, that is, maximal for unexpected events and minimal to frequent ones, irrespective of their magnitude. Functionally, predictive coding is a possible realization of the anticipatory function of the brain^1^,^4^,^5^. It assumes that the mismatch between descending and ascending information is assessed at each processing level, possibly at the cortical column scale^6^, so that only the error signal is further propagated. This theory is seducing because it offers a parsimonious computational mechanism to flexibly minimize stimulus-driven information, depending on the stage where stimulus features are anticipated, from sensory to high-level action representations^7^,^8^,^9^. The predictive coding framework potentially accommodates a number of well-known psychophysical and macroscopic neurophysiological phenomena, such as priming, repetition suppression and mismatch negativity^3^,^10^,^11^,^12^. At the biophysical and mechanistic levels, however, there are virtually no data showing how predictive coding could operate. Only a few theoretical proposals attempt to describe how predictions and prediction errors are computed and how information is being transferred in each direction^6^.

One of those stipulates that ascending and descending information could be conveyed via distinct frequency channels, the *γ*- and *β*-channels for up- and downgoing information, respectively^7^,^13^. This would imply that the brain uses multiplexing^14^,^15^ as a means to transmit signals of different nature and content in parallel and opposite directions. In particular, top-down (T-D) *β*-activity could provide a modulatory gain on lower-tier *γ*-activity^16^,^17^. In the context of predictive coding, a frequency dissociation between bottom-up (B-U) and T-D information transfer, with slower rates for T-D mechanisms, could be accounted for by the fact that T-D-propagated signals (predictions) follow from the linear accumulation of prediction errors^6^.

Experimental evidence of a spectral dissociation for up- and downgoing information remains scarce and not unequivocal. When probing two hierarchical regions of the monkey visual cortex during a spatial attention task, the *γ*-up/*β*-down scheme was only partly confirmed^18^. Moreover, even if spectral multiplexing would keep B-U and T-D information apart, a scheme with only two modulation bands (*β*and *γ*) and one carrier (high-*γ*) might be underspecified^19^.

Here we explored whether the human brain propagates B-U and T-D signals using distinct frequency bands. We used human depth electrode recordings made at locations corresponding to two successive steps in speech processing^20^ (Fig. 1). We examined the directionality of cross-regional interactions using non-parametric Granger causality (GC)^21^,^22^ to establish the dominant direction of information flow within specific frequency bands, and cross-regional phase-amplitude coupling to address whether high-frequency power in one region was modulated as a function of the phase of low-frequency activity in the other one (see Fig. 1a). By restricting cross-regional coupling results to those frequency domains where causality is significant, we show that B-U and T-D information transfer dominates in high- and low frequency-domains, respectively, and proceed in a discontinuous fashion, suggesting both frequency and time division are used to unmix up- and downgoing information in the brain.

## Results

### Characterization of intra-cortical responses to speech

**Figure 1:**
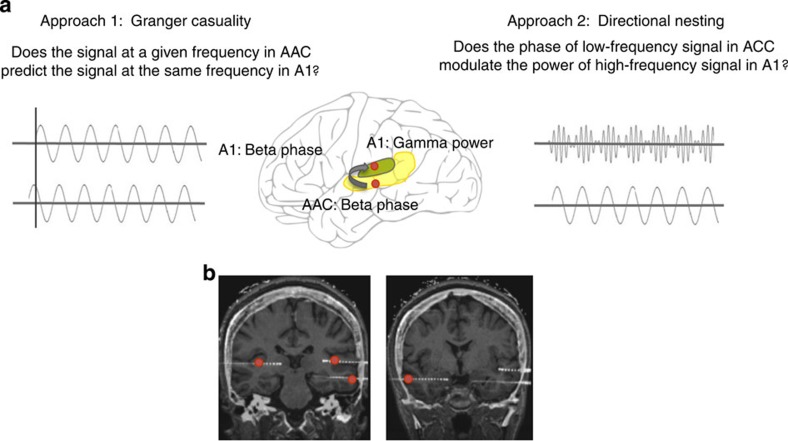
Experimental approach and electrodes position. (**a**) Experimental approach and hypotheses. We explored the processing of speech in auditory cortex through two distinct tests: GC, which allows for testing causal relationship between different regions within the same frequency band; and directional phase–amplitude coupling that examines phase-power dependencies both across brain areas and across frequencies. *β*- and *γ*-Frequencies were of particular interest in view of our working hypotheses. (**b**) Example of electrode positioning. In S1, electrodes were positioned at equivalent locations on each hemisphere in A1 and auditory association cortex (AAC). The electrode contacts used along the shaft were selected based on their anatomical location and functional responses (typical shape and latencies of evoked responses^66^).

**Figure 2:**
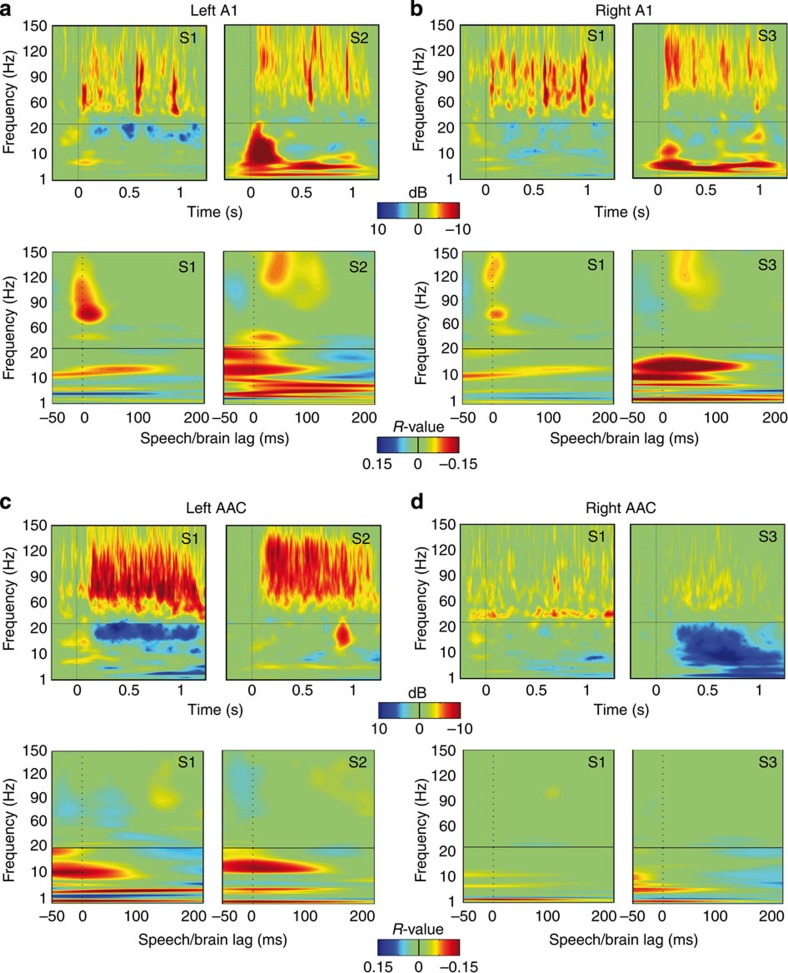
Individual regional speech-induced corticograms and speech/brain cross-correlation. Individual trial-averaged time-frequency representations (corticograms) of neural response to sentences (upper panels) and trial-averaged within-frequency speech/brain amplitude cross-correlograms (lower panels), computed in left (**a**) and right (**b**) A1, and left (**c**) and right (**d**) AAC. The dotted lines indicate sentence onset (upper panels) and zero-lagged speech/brain cross-correlation (lower panels).

We present results from three epileptic subjects who underwent a simple experiment during which they passively listened to spoken sentences. The protocol was part of a larger one involving more speech material (syllables and words)^23^ aiming at investigating speech-specific responses throughout the temporal cortex. We focused on the sentences data set and analysed intracortical activity from depth macroelectrodes located in primary (A1) and association auditory cortex (AAC) regions (Fig. 1b) in response to passive listening of 110 repetitions of 2.5-s long sentences. We assumed that spoken sentences had sufficient duration and complexity to engage B-U and T-D information transfer. One of the three subjects (S1) was implanted bilaterally in the two temporal lobes at identical functional locations (Fig. 1b). The two other subjects were implanted in the left (S2) or the right (S3) temporal lobe, for a total of two subjects per hemisphere and location. The limited number of subjects is inherent to the method as depth electrodes are rarely inserted in humans’ auditory cortex, and more rarely at equivalent functional sites in the left and right hemispheres. We analysed signals from the electrode contacts showing the most typical evoked electrophysiological landmarks of A1 and AAC (typical latencies and auditory-evoked response shapes; see Methods). The corticograms obtained for all subjects at each location confirmed distinct response patterns in A1 versus AAC regions (Fig. 2a–d, upper panels). Cross-correlating the wideband stimulus envelope^24^,^25^ with the cortical responses (see Methods) emphasized the functional distinction across hierarchical levels (Fig. 2a–d, lower panels). In A1 *γ*-activity correlated with the speech acoustic structure, whereas, consistent with a more integrated function of higher-level regions, *γ*-activity in AAC (left dominant) was largely induced and independent from fast acoustic modulations. Frequency-specific interactions between the acoustic speech signal and the intracortical electroencephalography (iEEG) were also observed in the *δ*/*θ*-band in all regions, and in the low *β*-band (around 12–14 Hz) in all regions except in the right AAC. Finally, in accordance with a weaker specialization for speech of right temporal regions, stimulus/brain cross-correlations were overall stronger in the left than in the right A1 (Fig. 2a,b, lower panels), and *γ*-induced responses in AAC were drastically left dominant (Fig. 2c,d, upper panels).

### Cross-regional GC

**Figure 3:**
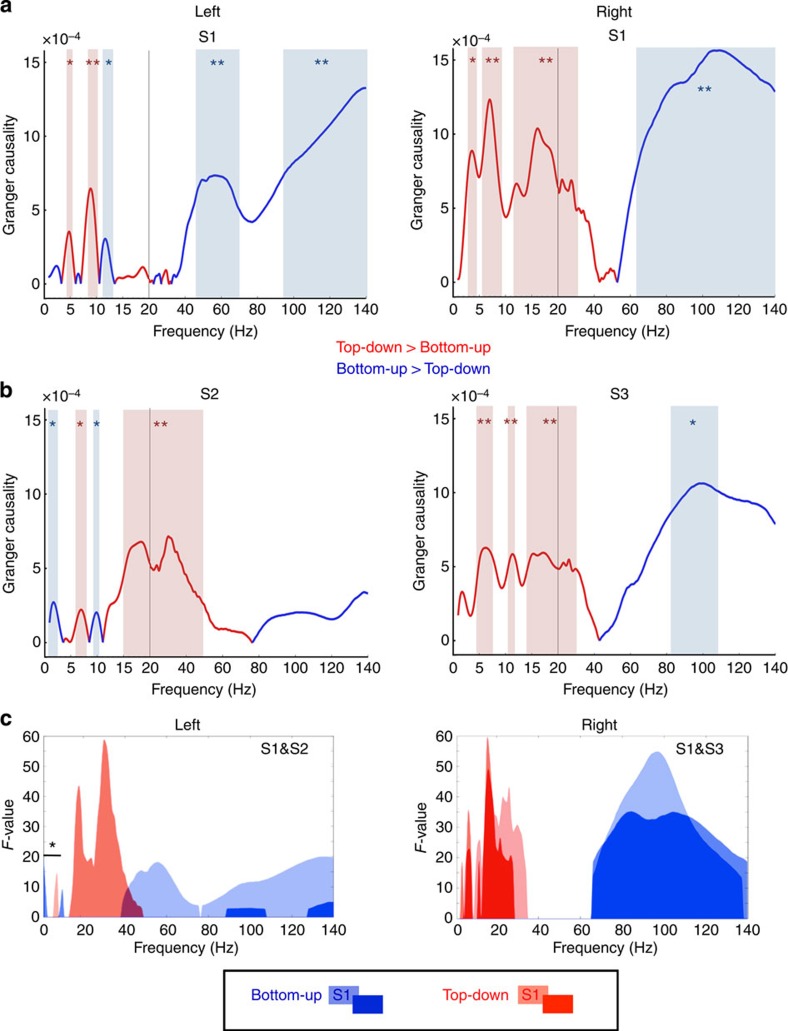
Spectral asymmetry between B-U and T-D using GC. Spectral differences (1–140 Hz) between T-D (AAC to A1) and B-U (A1 to AAC) causal influences in left and right auditory cortex of subject 1 (**a**) and subjects 2 and 3 (**b**). Red and blue lines show, respectively, T-D and B-U predominance, averaged over time, trials and sentences. Statistically significant differences between T-D and B-U are highlighted (shaded bars; FDR correction; *q<0.05, **q<0.01). (**c**) F-values obtained from one-way analysis of variance (ANOVA) analysis for each subject, testing the difference between T-D (red) and B-U (blue) causal directions. Data from S1 are shown in semi-transparent colours, and data from S2 and S3 in full colours. Only significant values are shown (*P*≤0.05, Bonferroni corrected). The star further indicates an interaction (two-way ANOVA, see Methods) in the 1–6 Hz range, where B-U GC dominates in the left hemisphere (*P*≤0.05, Bonferroni corrected).

We examined directed functional connectivity between A1 and AAC across the whole iEEG spectrum. We compared propagation directions by computing non-parametric GC in the frequency domain^21^,^22^ on a trial-by-trial basis for each subject (see Methods). We further averaged the GC time-frequency (TF) patterns across time, trials and sentences. In both the B-U (A1 causing AAC activity) and the T-D (AAC causing A1) analyses, we found several GC peaks distributed between 1 and 140 Hz (Supplementary Fig. 1 and Table 1 for details in the 1–20 Hz range). We observed that GC values were overall larger at low than at high frequencies. This global decrease in GC values might be related to the power law decay in the amplitude spectrum of brain activity^26^, as GC is sensitive to asymmetries in the power spectra. Critically, we found a dominance of T-D GC in the low part of the spectrum (<40 Hz) and of B-U GC at high frequencies (>40 Hz). This effect was confirmed using two complementary statistical approaches (Fig. 3a,b versus Fig. 3c), and the spectral division of B-U and T-D was consistent across subjects (and hemispheres). Although B-U dominance above 40 Hz is in line with the hypothesis that the brain mostly uses the *γ*-channel to propagate sensory information forward, T-D GC dominance was not limited to the *β*-range^6^,^7^,^13^, but broadly covered the whole *δ*–*β*-range. In the left hemisphere, we additionally found several discrete GC peaks in the B-U direction within the 1–20 Hz range. The B-U and T-D GC peaks did not align across subjects (Fig. 3, Supplementary Fig. 1, insets and Table 1), yet in each individual B-U and T-D directions appeared spectrally non-overlapping (Supplementary Fig. 1, insets): B-U GC peaks aligned to the troughs of T-D GC peaks and vice versa. Such a frequency splitting might indicate that specific sub-ranges within the *δ*–*β* domain (<40 Hz) specialize in directional information transfer, even though T-D overall dominates in this frequency range. Altogether, these results are consistent with the hypothesis that the brain uses distinct frequency channels to propagate feed-forward and feedback information. However, the picture arising from the GC data appears more complex than a simple *γ*-up/*β*-down scheme, and also involves lower frequencies^6^,^7^,^13^.

### Cross-regional phase-amplitude coupling

**Figure 4:**
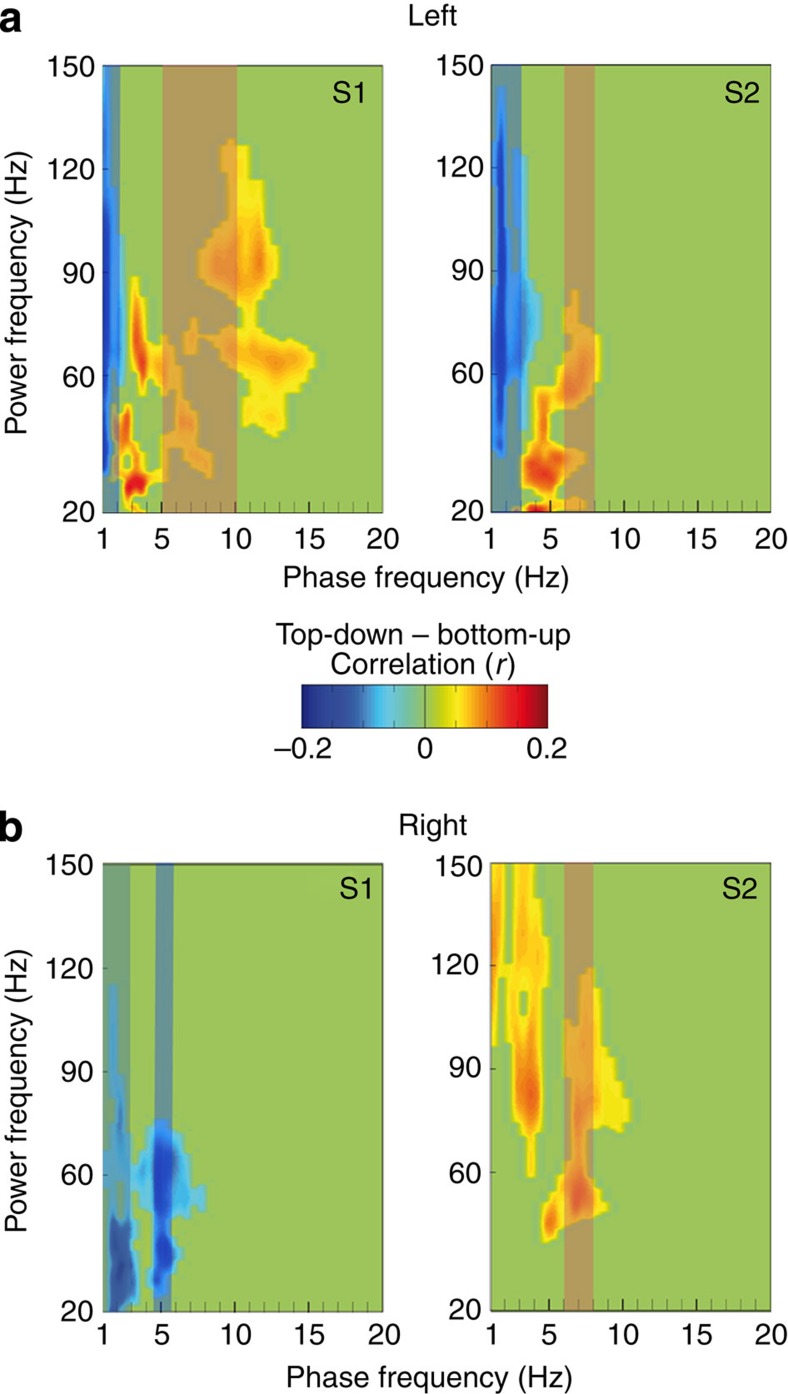
Cross-regional dominance of phase–amplitude coupling. Circular-to-linear correlations computed between low-frequency phase (1–20 Hz) of one region (A1 or AAC) and higher-frequency power (20–150 Hz) of the other region in left (**a**) and right (**b**) hemispheres. B-U (A1-phase modulating AAC power) and T-D (AAC-phase modulating A1 power) phase–amplitude coupling values were contrasted, with blue and red clusters indicating B-U and T-D dominances, respectively. In addition, we controlled that inter-regional cross-frequency dependencies dominated over local ones, by controlling for local phase, that is, performing the contrast (T-D—local-AAC)—(B-U —local-A1). Only significant (*P*≤0.01, cluster corrected) contrasts are reported. Shaded bars represent GC peaks (see Table 1) overlapping with phase–amplitude coupling results at the frequency of the modulating phase.

GC indicated the predominant direction of information transfer, but did not provide information about the influence ascending or descending signals may locally exert on neural activity. We therefore explored whether low-frequency T-D rhythms influenced distant (for example, AAC influence on A1) *γ*-power changes, reasoning that efficient information transfer should modulate the timing and/or the amount of population spiking at target level^27^,^28^. In particular, we tested whether the GC peaks observed in the low-frequency range (1–20 Hz) were associated with distant *γ*-power changes. To characterize the influence one region exerted on the region hierarchically below or above, we examined cross-regional nonlinear coupling across frequency bands, as the excitability of neuronal populations in sensory systems is shaped by low-frequency oscillations through their phase^29^,^30^. To do so, we computed circular-to-linear correlations that quantify how the phase of low frequencies sampled in one region co-varies with the amplitude of higher frequencies in the other region. We confirmed that cross-regional effects were stronger than local ones (Fig. 4), that is, within-region phase-amplitude coupling (see Supplementary Fig. 2 and see Methods). Statistical significance of phase-amplitude coupling was assessed using a non-parametric cluster analysis^31^ (see Methods). For each significant cross-regional phase-amplitude coupling cluster, we subsequently confirmed that there was a corresponding GC peak at the frequency of the modulating phase (Table 1). In Fig. 4, vertical shaded bars indicate the overlap of phase-amplitude coupling and GC peaks. Overall this ensures that the observed modulations of *γ*-power were driven by the phase of distant lower frequencies.

Consistent with the notion that T-D information propagated in the low-frequency range has an influence on local *γ*-activity, we found a modulation of *γ*-power in the left A1 as a function of the phase of *θ*-activity in the left AAC in both subjects (Fig. 4a and Supplementary Fig. 2). This pattern was similar in the right hemisphere, even though it survived statistical correction only in S3 (Fig. 4b). To each T-D (AAC-phase/A1-power) phase-amplitude coupling cluster corresponded a T-D GC peak at the phase frequency (Fig. 4, shaded red bars; see Table 1), allowing to conjecture that the T-D influence of AAC on A1 observed with GC in the low-frequency range was associated with *γ*-power modulation at target level. We also detected a significant modulation of *γ*-power in AAC as a function of the phase of *δ* (1–3 Hz) activity measured in A1. As for T-D effects, B-U GC peaks (Table 1) aligned with each of these clusters (Fig. 4, shaded blue dotted bars). Finally, we observed a left dominance of this effect at *δ*-rate, both with GC (Fig. 3c) and phase-amplitude coupling (Supplementary Fig. 3) measures, suggesting that B-U flow was stronger at very low frequencies (1–2 Hz) in the left hemisphere.

Altogether, GC and phase-amplitude coupling measures concurred to show a frequency division for B-U and T-D information transfer, whereby local *γ*-activity was globally modulated as a function of distant *δ*-phase in the B-U direction (Fig. 4, blue clusters) and as a function of distant *δ–β* phase in the T-D direction (Fig. 4, red clusters). These findings suggest that the multiplexing of B-U and T-D information transfer operates, at least in part, by varying the modulation frequency of local *γ*-activity.

### Time division in B-U and T-D causality

**Figure 5:**
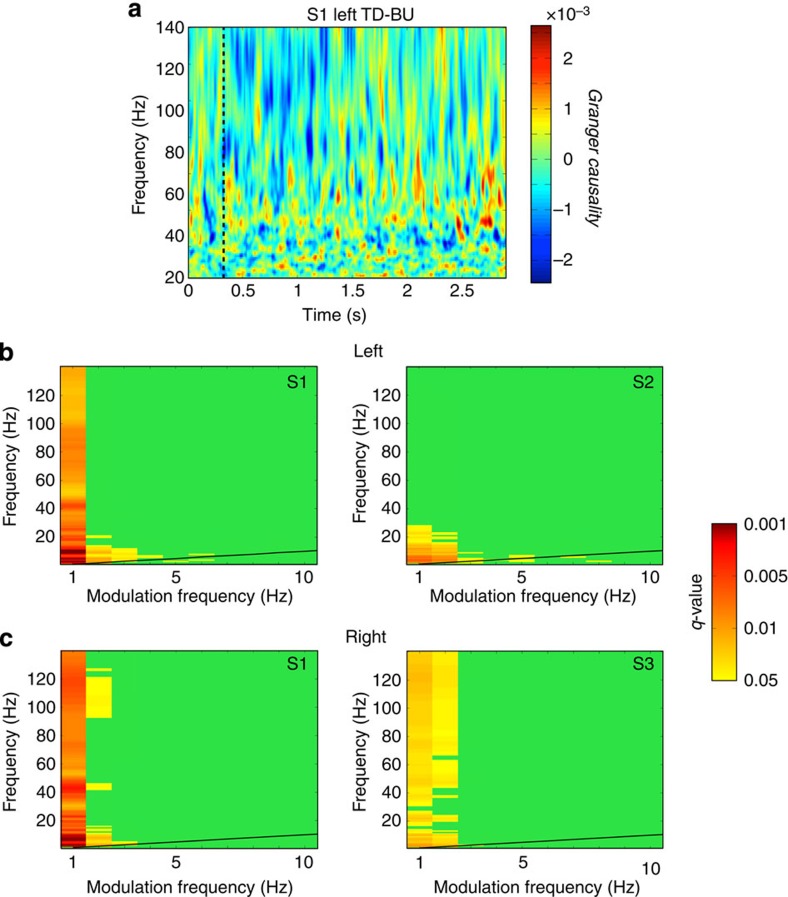
Temporal modulations of GC. (**a**) Difference between T-D and B-U Granger causal influence plotted as a function of time and frequency (shown for S1). GC time–frequency representations do not show evidence of continuous causal influences across distinct frequency bands, for example, B-U *γ* and T-D *δ–β*. Conversely we observe alternations in both time and frequency, suggestive of a discontinuous pattern of information transmission. GC modulation spectrum in left (**b**) and right (**c**) auditory cortex, resulting from Fourier-transforms of GC time–frequency data. Only significant (*q*≤0.01, FDR corrected) modulations are reported. Black lines correspond to the diagonal (that is, modulation frequency=modulated frequency).

Directional multiplexing by spectral division enables continuous information transfer in B-U and T-D directions simultaneously. To assess whether information transfer was indeed continuous in both directions (and related frequency ranges), we computed GC at any instant (1 ms resolution) during the processing of auditory sentences to obtain T-D and B-U GC TF representations. Contrary to what we expected, we did not observe lasting periods when GC dominates in one direction or the other. Rather, TF GC was organized as an alternation of frequency-specific bins, suggesting that information transfer proceeds by alternating periods of dominant B-U and dominant T-D (Fig. 5a, cold colours for B-U and hot colours for T-D). We tested for a periodicity in the alternation of T-D and B-U GC by computing the Fourier spectrum of T-D minus B-U TF matrix (Fig. 5b,c; false discovery rate (FDR) correction, see Methods). Although B-U and T-D flow did not use the same frequency ranges, we found a common low rate temporal arrangement. In all subjects, a significant temporal modulation at *δ*-rate (1–3 Hz) was observed (Fig. 5b,c; see permutation tests in Methods). The results hence suggest an alternation of dominant T-D and B-U information transfer approximately every 300–500 ms in both flow directions. It is important to note that the temporal structure of GC changes may also reflect that GC is based on a linear model of temporal dependencies, which cannot account for the nonlinear dependencies we have established in terms of cross-regional phase-amplitude coupling. It is hence unclear whether this alternation would hold for inter-regional cross-frequency coupling effects.

## Discussion

In telecommunications, frequency-division multiplexing refers to the use of separate carrier frequencies to transmit distinct modulated signals on a single physical support^32^. In auditory processing, the notion of multiplexing is increasingly referred to in a different acceptation, that is, the description of parallel information processing at several timescales^14^,^15^. Here we explored the notion that the brain uses directional multiplexing, whereby B-U and T-D information are propagated using distinct modulation frequencies and/or different carrier frequencies. This idea arises from neurophysiological studies showing that the low-*β*-range (around 14 Hz) was mostly related to endogenous T-D process^33^,^34^,^35^,^36^. From a theoretical viewpoint, the idea that the brain constantly compares incoming input with internal representations^1^,^5^, calls for information processing at different timescales^6^,^7^,^13^ and information unmixing.

The current results indicate a frequency dissociation in information transfer along the auditory cortical hierarchy: *δ–β* frequencies dominated in the T-D direction and *γ*-frequencies in the B-U direction. These findings support theoretical proposals^6^,^13^ and complement other results—so far only partly conclusive—obtained in humans and monkeys^18^,^33^,^34^,^35^,^36^,^37^. By combining GC and nonlinear cross-regional phase-amplitude coupling, we further show that T-D processes operated by modulating fast neural activity at *δ–β* rates in the target area, that is, the *γ*-power in A1 being modulated as a function of the phase of low frequencies in AAC. Furthermore, we observed several T-D GC peaks in the *δ–β* (4–30 Hz) frequency range, and the frequency of these peaks varied across individuals (and hemispheres). Overall, we detected more GC peaks than directional phase–amplitude coupling clusters, suggesting either that not all the detected GC peaks translated into phase-power modulations between source and target locations, for instance due to the presence of phase–phase coupling^38^, or that the circular-to-linear correlation method was not sensitive enough to detect them all. Importantly, however, to each single phase–amplitude coupling cluster corresponded a GC peak. This cross-validates the results and ensures that *γ*-modulations by the phase sampled from another region truly reflects distant modulations. Combining GC and phase–amplitude coupling constitutes an exploratory alternative to dynamic causal model (DCM)^39^. Chen *et al*.^40^ used DCM to assess amplitude–amplitude cross-frequency coupling between high- and low-visual areas during perception of human faces. They also found qualitative evidence for functional asymmetry coupling, where the effects of low frequencies on high frequencies were greater in the backward direction relative to the forward direction. However, although DCM allows for an exploration of linear and nonlinear interactions within a single model, our approach may be more flexible in discovering non-hypothesized neurophysiological phenomena, such as those we detected in the very low-frequency range (see ref. 41 for a comparison of DCM and GC).

Our findings confirmed that T-D neural flow uses lower frequency ranges than B-U, but also point to a more complex picture than the previously hypothesized *γ*-up *β*-down scheme^6^,^18^,^33^,^34^,^35^,^36^,^37^. In left auditory regions, we detected GC B-U peaks in the *δ*-frequency range (1–3 Hz), indicating that very low frequencies were also involved in B-U transfer (Table 1). Cross-frequency coupling did not only confirm this effect but further showed that the phase of *δ*-activity sampled in left A1 was associated with a modulation of high *γ*-power (80–100 Hz) in left AAC (Fig. 4). The fact that this effect dominated in the left hemisphere (Supplementary Fig. 3) could reflect (i) that *γ*-activity in AAC was more pronounced in left than right AAC (Fig. 2), (ii) that the low-frequency phase-locking of responses (Supplementary Fig. 4) and stimulus/brain correlations (Fig. 2) were stronger in left than right A1, (iii) or both. At any rate, the left dominance in *δ*/*γ*-coupling presumably reflects some aspects of the functional specialization of left auditory regions in speech processing^42^. Importantly, the spectral division of B-U and T-D information transfer appears partly flexible, depending on cognitive demands and/or on the interaction of stimulus rhythms with local oscillatory properties.

The current results do not only show a spectral division but also suggest a time division of labour between B-U and T-D processes. The analysis of temporal modulations of GC TF representations (Fig. 5) revealed the presence of significant slow fluctuations (1–3 Hz) of GC in the *γ*-frequency range, suggesting that B-U and T-D information sequentially dominated over periods of ~300–500 ms. What could determine the regular alternation of B-U- and T-D-dominant periods at this slow rate remains unclear at this point. It could result from time constants that are specific to speech. It has been shown that predicting forthcoming speech involves a syllable-based mechanism^43^, which is roughly compatible with 300 ms predictive segments. Alternatively, slow modulations could be entirely driven by endogenous *δ*- or *α*-rhythms, whose phase (i) determines whether a stimulus is going to be detected at the sensory level^44^,^45^,^46^ and (ii) indexes the dynamics of sequential information processing at higher stages^47^. In this respect, it would be interesting to obtain similar data from the visual modality with less temporally structured stimuli, and assess to what extent the timing of GC is stimulus driven, or emerges from properties of the brain organization.

The main advantage of spectral multiplexing is to prevent interferences during multiple and continuous information transfer using the same physical support. In the cortex, BU and TD information travel via separate vectors^13^ but information unmixing, by, for example, multiplexing, is required in relays where ascending and descending information converge and are integrated (that is, superficial layers). The modulation of neuronal spiking (here approximated by local high *γ*-activity^28^) at distinct, *δ–β* versus *γ*, rates could be an efficient means to achieve it. Alternatively, interferences could be avoided by using a single information channel, with time windows during which B-U or T-D dominates. Although the observed spectral division between B-U and T-D information flows (Fig. 3) supports the former scenario, temporal alternation of ascending and descending information (GC modulations; Fig. 5) supports the latter scenario. Although frequency and time division are not conceptually incompatible, the use of both mechanisms for directional information transfer appears computationally redundant. The presence of effects in the *δ* (1–3 Hz) range for both spectral (Figs 3 and 4) and time (Fig. 5) division is fairly compatible with the predictive coding framework. This reflects the fact that both B-U and T-D tend to fluctuate at *δ*-rate (Fig. 5), while being predominantly oriented towards the B-U direction (Fig. 3 and Supplementary Fig. 3). Such an asymmetry in information flow is in line with the proposal that T-D message passing results from the accumulation of B-U evidence, as this process could translate in the co-occurrence of a continuous B-U accumulation of prediction errors and a discontinuous T-D prediction flow. More generally, the present results are consistent with predictive coding models in the sense that T-D predictions of auditory input rest on a nonlinear mapping from higher-level representations, as shown by the nonlinear cross-regional phase–amplitude coupling results (Fig. 4).

T-D effects were mainly associated with modulations of *γ*-activity at rates ranging from 5 to 30 Hz. The *β*-rhythm could have a local (cortical) origin that is compatible with a function in T-D control. An interesting model of *β*-generation from *in vitro* slice preparations suggests that low *β*-activity could result from the local concatenation of two independent higher-frequency rhythms (*γ*) generated in superficial and deep layers, respectively^48^. *In vitro* experimental observations^48^,^49^ indicate that there is an alternation between either the two independent higher-frequency rhythms or the *β* one, depending on the level of excitation and on synaptic plasticity. We previously speculated that this generation mode could constitute a possible mechanical switch between a state where information flows freely upward (*γ* in deep and superficial layers) and a state where information transfer is redirected, first confined within a cortical level and then directed downward^7^. From an information-processing perspective, the latter state could serve to match ascending and descending information, and reduce the discrepancies between incoming inputs and internal representations. Remarkably, the biophysical mechanism of *β*-generation proposed by Roopun *et al*.^49^, involving the concatenation of slow and fast rhythms, implies the temporal alternation of B-U dominant phases and T-D dominant phases, consistent with what we observed here.

An alternative account for the frequency range where T-D effects operate could be related to the *α*-rhythm physiology, as T-D phase–amplitude coupling effects were largely distributed around 10 Hz. *α*-Rhythm is the most conspicuous and widespread of the brain rhythms owing presumably to its thalamo-cortical origin^50^, and is an important effector of attentional processes^51^,^52^. Although it could be involved in descending mechanisms, its general role in attention and sensory gating is hardly compatible with one in specific information and representations transfer. However, *α*-rhythm displays bistability with occasional splitting in high-*θ* and low-*β* components^53^,^54^,^55^. It is unclear how this splitting operates, but if it was associated with a change from a widespread generation mode to a more local one, it could also possibly underpin the effects we observe here. Alternatively, low *β*-oscillations could be generated by the interaction between a feedforward and a feedback *α*-input, as suggested by another model^56^.

Thanks to unique depth intracortical human recordings collected in two hierarchical regions of speech processing, bilaterally in one subject and unilaterally in other two subjects, we show a spectral division of the B-U and T-D processing flow. Although our findings confirm a *γ*-up scheme, they do not support a *β*-down one. They show that a larger band involving *δ–β* frequencies are involved in T-D information transfer, and further suggest that local *γ*-activity is modulated by the phase of lower-frequency distant oscillatory activity. We additionally showed that directional information transfer does not proceed continuously, but alternates at a 1- to 3-Hz rate. These data suggest that speech processing uses both distinct modulation frequencies and temporal windows to transfer information in B-U and T-D direction. The reason why B-U and T-D unmixing appear implemented in a redundant manner, and the extent to which the time constants we observed here are specific to speech processing or could instead generalize, remain open questions.

## Methods

### Subjects

Three French female subjects participated in this study. They suffered from drug-resistant partial epilepsy and were implanted for presurgical investigation with chronic depth electrodes in: right and left auditory cortex (S1, 45 years old), left auditory cortex (S2, 30 years old) and right auditory cortex (S3, 35 years old). The electrodes of interest were located in Heschl’s gyrus (primary auditory cortex, A1) and laterally in the superior temporal gyrus in a region we refer to as AAC, as well as in other cortical structures that were not relevant for the study (see functional validation of electrode position below). The subjects provided informed consent to the protocol, which was approved of by the institutional review board of the French Institute of Health. Neuropsychological assessment indicated that they had intact language functions. Brainstem-evoked potentials and pure tone audiograms carried out before iEEG indicated intact cochlear and brainstem auditory functions. Analysis of iEEG indicated that the epileptic zones were located outside the regions examined here.

### Electrophysiological recordings

*Stimuli and data acquisition.* The subjects listened to 110 repetitions of two 2.5-s-long sentences in French, uttered by a French female whose voice had a fundamental frequency of 201 Hz. Stimuli were presented monaurally to both ears, in a pseudo-randomized order with an interstimulus interval of 4,135 s, and only the contra-lateral response was taken into account. The stimuli were recorded digitally at a sampling frequency of 44.1 kHz and delivered to the subjects at 22 kHz with a 75-dB sound pressure level headset using E-prime software (Psychology Software Tools Inc., Pittsburgh, PA, USA).

iEEG recordings were monopolar, with each contact of a given depth electrode referenced to an extra-dural lead using acquisition software and a 128-channel SynAmps EEG amplification system from NeuroScan Labs (Neurosoft Inc.). During the acquisition, the EEG signal was high-pass filtered at 0.5 Hz and amplified with an anti-aliasing filter at 200 Hz (temporal resolution of 1 ms and amplitude resolution of 1 μV).

### Anatomo-functional definition of contacts position

The stereotactic method was based on the co-registration of the subjects’ magnetic resonance imaging (MRI) with the stereotactic angiogram, to prevent injury to brain vessels. Multi-lead electrodes (0.8 mm diameter, 10 or 15 contacts of 2 mm length each with 1.5 mm spacing between contacts) were orthogonally introduced in the stereotactic space. The anatomical position of each contact was then identified on the basis of (i) an axial scanner image acquired before the removal of electrodes and (ii) an MRI scan performed after the removal of electrodes (see Fig. 1b for electrode position in S1). Auditory-evoked potentials measured in response to pure tones were used to functionally delineate A1 and AAC, and select the right electrodes. Auditory-evoked potentials were averaged over trials, after epoching (200–635 ms) and taking the 150–50 ms pre-stimulus time period as baseline. All contacts that elicited no significant responses were discarded. In a second step, A1 was functionally defined based on the presence of early P20/N30 components. These responses were located in the medial and intermediate part of Heschl’s gyrus. Third, for each functional area (A1 and AAC in left and/or right hemispheres), the most responsive contact was selected for subsequent analyses.

### Preprocessing

Data analysis was performed with EEGlab v.8 (sccn.ucsd.edu/eeglab) for data extraction, Fieldtrip (http://www.ru.nl/donders/fieldtrip) and Fast_tf (http://cogimage.dsi.cnrs.fr/logiciels/), for TF decomposition. For GC, we modified and adapted the scripts used in ref. 57. Data were epoched into segments, including a baseline period (328 ms for S1, 1000, ms for S2 and S3) before stimulus onset and an after stimulus period (452 ms for all subjects). Epochs including signals that deviated from the average response of all the trials were discarded, by computing the correlation between each single trial and the average response, and then rejecting the 15% of trials with the lowest Pearson’s correlation value. We set this conservative rejection criterion and validated the approach on the basis of visual inspection of the signals. The data set analysed was hence free of suspicious electrical activity related to epilepsy, for example, interictal spikes.

We used a bipolar montage for data analyses, meaning that electrical activity from all contacts was subtracted from a common reference signal that corresponded to the average response of the least-responsive (mesial or lateral) contact of each electrode. This resulted in attenuating global noise (50 Hz ambient electric field) in a similar way for all contacts.

### TF analyses and power spectrum

A TF continuous wavelet transform was applied to each epoch using a family of complex Morlet wavelets (*m*=7), resulting in an estimate of power and phase at each time point and each frequency, with a 0.5-Hz resolution below 20 and 1 Hz above. The TF resolution of the wavelets was frequency dependent (at 7 Hz: *σ*=150 ms, 1 Hz; at 35 Hz: *σ*=30 ms, 5 Hz). We restricted the analysis to frequencies between 1 and 150 Hz, spanning the whole range of relevant brain rhythms (up to high *γ*-activity). Figure 2 shows the typical cortical responses (increase or decrease in signal power relative to baseline in decimal logarithmic units (dB) at each time and frequency data point) for all subjects and brain areas.

### Speech/brain cross-correlations

*Sentences characterization.* For each sentence, we estimated the wideband envelope of the speech waveform^24^,^25^. The raw speech waveform was band-pass filtered into 32 frequency bands, encompassing 80–8,500 Hz with a logarithmical spacing, modelling the cochlear frequency decomposition. The absolute value of the Hilbert transform of each band-passed signal constitutes an estimate of the envelope for that frequency band, and their sum an estimate of the wideband envelope. Finally, we computed the power in each frequency band at each time point, with a millisecond resolution, similar to the iEEG data, that is, between 1 and 150 Hz, with a 0.5Hz resolution below 20 Hz and 1 Hz above, by applying a TF wavelet transform, using a family of complex Morlet wavelets (*m*=7).

*Speech/brain cross-correlation computation*. We cross-correlated over time for each trial, sentence and region the oscillatory power estimates of the neural data at each frequency (1–150 Hz) with the corresponding frequency of the acoustic signal, between −50 and 200 ms, relatively to the acoustic input (with brain response following acoustic signal for positive cross-correlation values). This procedure results in an estimate of stimulus/response correlation at each frequency and for multiple time delays. At each time delay, correlation equals 1 if the two signals are perfectly identical when taking into account this time delay, and 0 if the two signals are totally unrelated. Data were subsequently averaged over trials and sentences.

### GC analysis

GC is classically used to assess causal influence among two time series^22^. The basic assumption is that a time series *X*(*t*) linearly causes another time series *Y*(*t*) if the future trend of the latter is better predicted by looking at the past of *X* and *Y* than by looking at the past of *Y* alone. For stationary processes, the computation of GC relied on multivariate autoregressive models to estimate the prediction error in the two conditions^22^. For non-stationary time series, such as oscillating neural signals, GC spectra can be obtained in a non-parametric manner by computing Geweke’s frequency domain version of GC without going through the multivariate autoregressive model fitting^58^,^59^. We therefore used a spectral density matrix factorization technique on complex cross-spectra, obtained from the continuous wavelet transform of recorded iEEG time series^58^. Both parametric and non-parametric GC have been previously used in neuroscience to assess linear directional influence between two communicating brain areas in local field potential^60^, EEG^57^ and functional MRI data^61^.

GC was computed on a trial-by-trial basis for each subject using the method proposed by Dhamala *et al*.^58^, and then averaged across time, trials and sentences (Fig. 3 and Supplementary Fig. 1). Statistics were then computed by generating 1,000 permutations of iEEG data, in which left and right electrodes, as well as trials, were randomized. This procedure permits to rule out that the observed effects arose from noise or specific methodology, as the exact same data and algorithms were used to compute the permuted trials. For each realization, we computed the mean GC across trials and the corresponding s.d. The original GC spectra were then standardized to obtain a vector of *Z*-values, one for each frequency.

T-D and B-U influences can be measured simultaneously^18^,^62^. The information flow was considered T-D when GC from AAC to A1 exceeded GC from A1 to AAC, and B-U in the other case.

We tested for significant frequency peaks separately for each T-D and B-U GC direction (Supplementary Fig. 1 and Table 1) together with frequency ranges where T-D and B-U GC spectra were significantly different (Fig. 3a,b). For the first analysis, we directly compared the *Z*-transformed vectors obtained from GC spectra to a zero-mean normal distribution, and corrected for multiple comparisons with the FDR method at a one-tailed *q*-value of *q*≤0.05. For the second analysis, we first computed the difference in *Z*-values between T-D and B-U Granger spectra at each frequency point, and then compared it with the zero-mean normal distribution thresholding at a two-tailed *q*-value of *q*≤0.05 (or *q*≤0.01, see Fig. 3), FDR corrected.

To further explore effects of flow direction, we applied a one-way analysis of variance test on time-averaged GC spectra for all data sets at each frequency point (Fig. 3c). Red (blue) areas correspond to frequencies where the T-D (B-U) mean is significantly higher than the B-U (T-D) mean. Values were thresholded at *P*≤0.05 (Bonferroni corrected for multiple comparisons). We also performed a two-way analysis of variance to investigate together the effects of hemisphere (left/right) and flow direction (T-D/B-U). The four data sets were tested pairwise (contrasting S1 left and right hemispheres, and S2 with S3) and the values were thresholded at *P*≤0.05 (Bonferroni corrected for multiple comparisons). A significant interaction was detected at low frequencies (1–6 Hz).

To assess the temporal alternation of directional GC peaks, we first subtracted T-D and B-U TF matrices at each trial (see example in Fig. 5a). We then Fourier transformed this data at each frequency band (1 Hz resolution) and averaged across trials and sentences to obtain the modulation spectrum for each data set. For statistical testing, we computed 1,000 permutations of GC data by shuffling trials and electrodes, *z*-scored the data using the mean and variance obtained from permutations and used FDR correction for multiple comparisons (Fig. 5b,c).

### Cross-regional phase–amplitude coupling

Cross-frequency coupling dependencies were studied under the phase–amplitude coupling framework. The rationale for using phase–amplitude coupling is that cross-frequency interaction could provide dynamic gating of information. Cross-regional phase–amplitude coupling would in turn reveal direct nonlinear interactions between distant sites^63^. We used phase and squared power values (amplitude) to approximate circular and Gaussian distribution, respectively. We subsequently computed the circular-to-linear correlation^64^, between each 1–20 Hz phase and 20–150 Hz amplitude frequencies. Correlations were computed across trials, sentences and time dimensions altogether, resulting in an estimate of the amount *r* of correlation between two frequencies, under a phase–amplitude dependency. To compute inter-regional dominance, we contrasted T-D and B-U phase–amplitude coupling analyses, while controlling for their dominance over local effects. We controlled for local phase ((T-D—local AAC)—(B-U—local A1)) to ensure that the amplitude modulations detected in one region are significantly more strongly related to the low-frequency phase sampled in the distant region than to local low-frequency phase.

Significant phase–amplitude coupling was based on corrected *P*-values using non-parametric permutation tests to generate null distributions of the maximum cluster size^31^. This implicitly adjusts for searching over multiple frequencies. The null distribution was obtained by computing 1,000 times the circular-to-linear correlation from a random mix of data taken equitably from the four types of phase/power relations we investigated (T-D, B-U, local A1 and local AAC). Clusters were defined as contiguous *r*-values above 0.045. Clusters of the 99th percentile (corresponding to *P*≤0.01) were considered significant and are reported throughout the manuscript.

To further explore effects of flow direction and hemisphere (Supplementary Fig. 3), we contrasted the left and right phase–amplitude coupling patterns observed in Fig. 4 (T-D minus B-U corrected for local phase). To highlight only left-dominant results, we applied to the left–right contrast a mask corresponding to the left hemispheric phase–amplitude coupling patterns. Statistics were computed similarly than before, except that we took a random mix of data taken equitably from the eight types of phase/power relations we investigated (left/right, T-D/B-U, local A1/AAC). In this approach, clusters were defined as contiguous *r*-values above 0.07 and clusters of the 99th percentile (corresponding to *P*≤0.01) were considered significant.

### Phase-locking value

For each region of interest, we evaluated the evoked modulation spectrum. We computed the phase-locking value^65^ across trials for each time point and 1–150 Hz frequency. To obtain the modulation spectrum, we averaged the resulting phase-locking values over time and sentences.

## Additional information

**How to cite this article:** Fontolan, L. *et al*. The contribution of frequency-specific activity to hierarchical information processing in the human auditory cortex. *Nat. Commun.* 5:4694 doi: 10.1038/ncomms5694 (2014).

## Supplementary Material

Supplementary Information Supplementary Figures 1-4

## Figures and Tables

**Table 1:** Low frequency peaks in Granger causality.

**Frequency peaks**	**Top-down**				**Bottom-up**			
	**Peak 1**		**Peak 2**		**Peak 1**		**Peak 2**	
	**Hz**	***P*** **-value**	**Hz**	***P*** **-value**	**Hz**	***P*** **-value**	**Hz**	***P*** **-value**
S1 Left	5	0.05	9	0.01	1	0.05	7	n.s.
S2 Left	7	n.s.	13	n.s.	2	0.05	10	n.s.
S1 Right	7	0.01	16	0.05	2	0.05	5	n.s.
S3 Right	7	0.01	12	n.s.	3	n.s.	9	n.s.

n.s., non-significant.

Low-frequency (1–20 Hz) peaks (2 maximum) for each data set and causal direction (top-down or bottom-up; excerpted from Supplementary Fig. 1. Peaks are sorted in ascending frequency, shown under the Hz columns, with the corresponding significance level (see Methods) shown under *P*-value columns (statistics are FDR corrected).
