# Supplementary material for: The contribution of frequency-specific activity to hierarchical information processing in the human auditory cortex
Source: Nat Commun. 2014 Sep 2;5:4694. doi: 10.1038/ncomms5694 (PMC4164774; doi:10.1038/ncomms5694)
Supplement: Supplementary Information — Supplementary Figures 1-4 [file ncomms5694-s1.pdf]

SUPPLEMENTARY INFORMATION

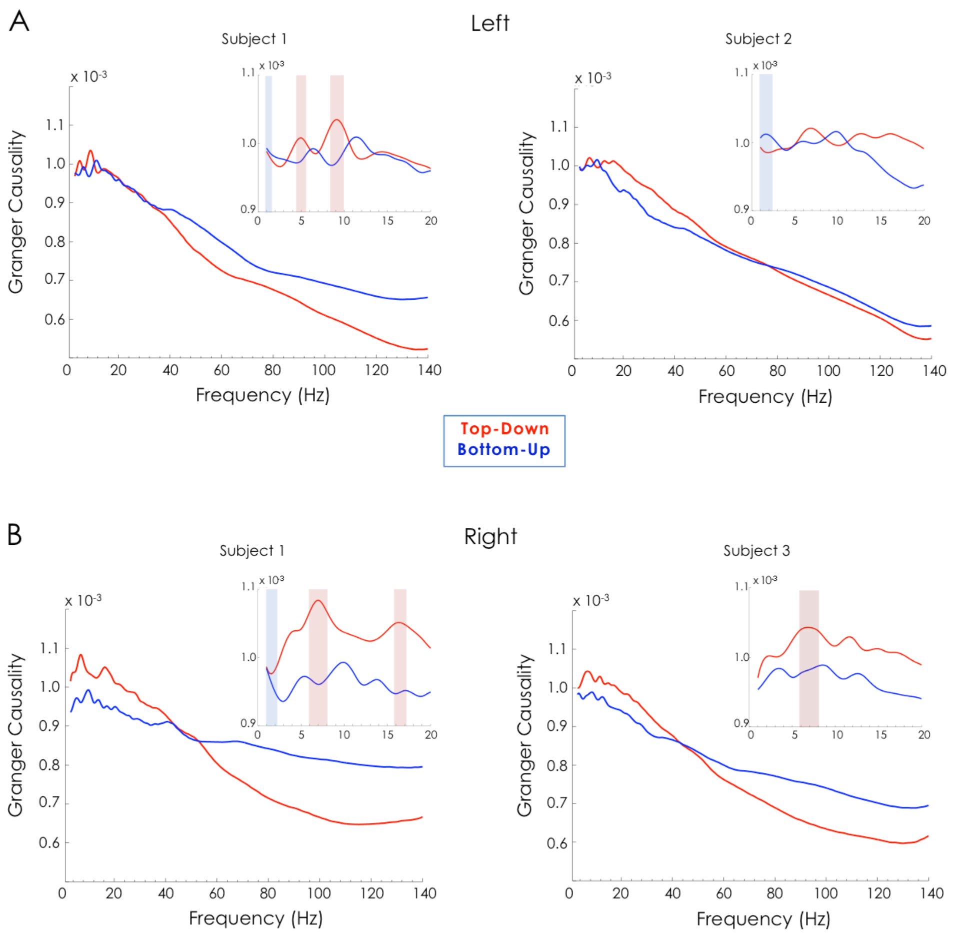

Supplementary Figure 1. Main effects of Granger causality. Spectral patterns (1-140 Hz) of top-down (red) and bottom-up (blue) causal influences in left **(A-B)** and right **(C-D)** auditory cortices, averaged over time, trials and stimuli. *Inset:* zoom on low (1-20 Hz) frequencies. Statistically significant peaks in each T-D or B-U causal direction are highlighted (shaded bars; FDR correction, $q \leq 0.05$).

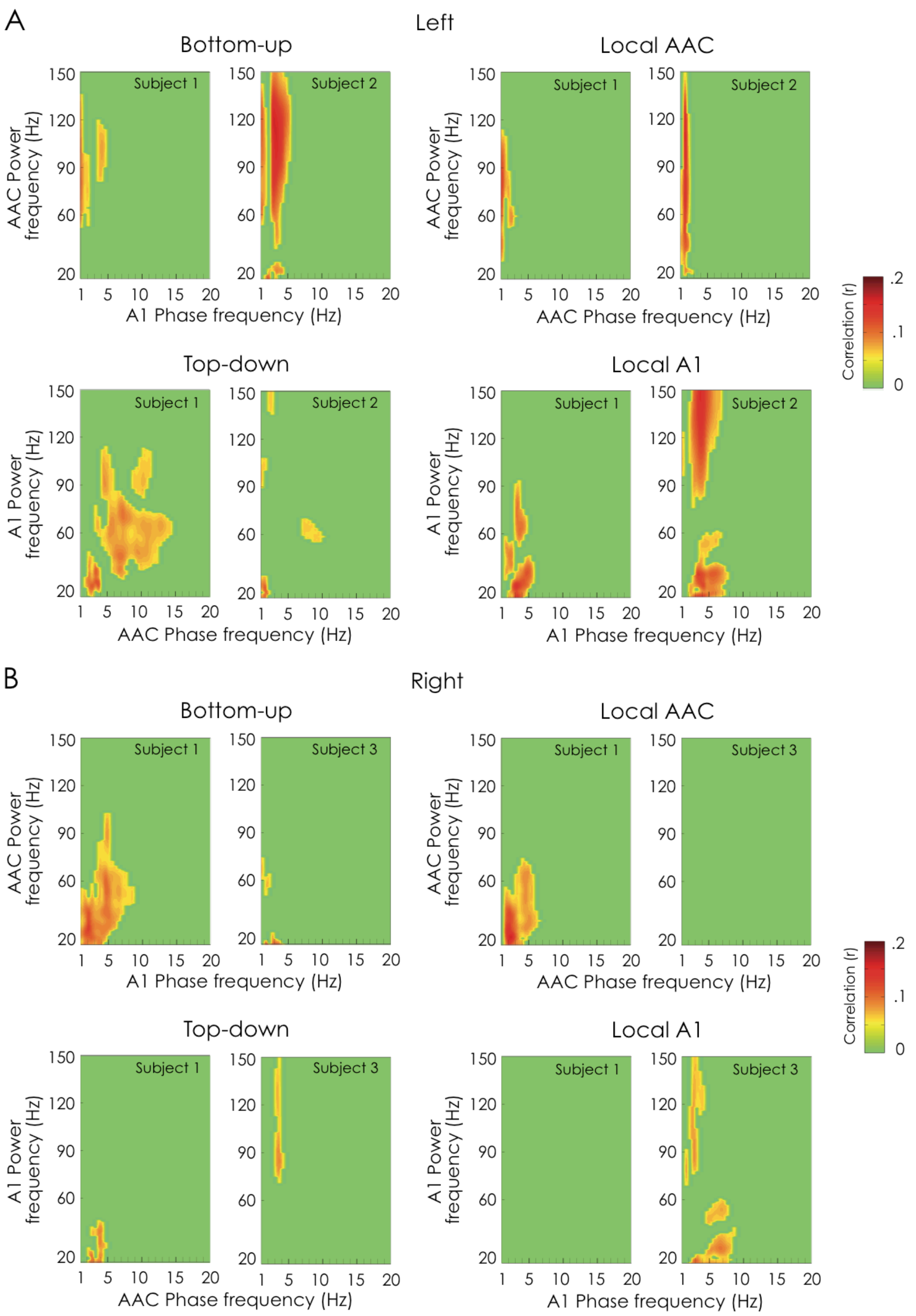

Supplementary Figure 2. Main effects of phase-amplitude coupling. Circular-to-linear correlations computed between low-frequencies phase (1-20 Hz) and high-frequencies power (20-150 Hz) in left **(A)** and right **(B)** hemispheres for each subject. *Upper left panel:* Bottom-up coupling (A1-phase modulating AAC-power). *Upper right panel:* Top-down coupling (AAC-phase modulating A1-power). *Upper and lower right panels:* Local coupling in AAC and A1, respectively. Only significant ($p \leq 0.01$, cluster corrected) values are reported.

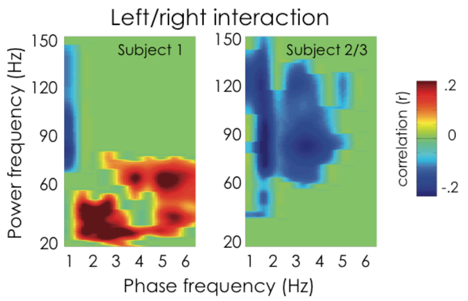

Supplementary Figure 3. Laterality effects of phase-amplitude coupling. Circular-to-linear correlations computed between low-frequencies phase (1-20 Hz) and higher-frequencies power (20-150 Hz). *Left* and *right* hemispheric nesting values obtained in Figure 4 were contrasted, and further masked with the nesting values of the left hemisphere (Fig. 4A), to highlight left-dominant effects. Blue and red clusters thus indicate *bottom-up* (A1-phase modulating AAC-power) and *top-down* (AAC-phase modulating A1-power) left dominances, respectively. Only significant ($p \leq 0.01$, cluster corrected) contrasts are reported.

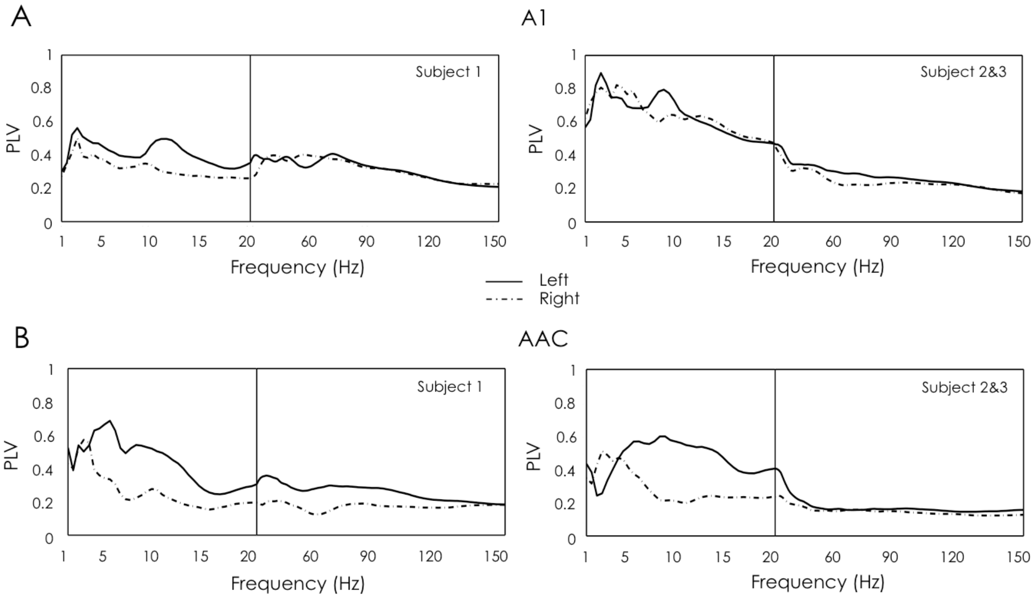

Supplementary Figure 4. Evoked responses spectrum. Phase-locking values (PLV) computed across trials between 1-150 Hz and averaged over time and stimuli. Left and right evoked spectrums are juxtaposed for A1 **(A)** and AAC **(B)** regions.
